# Assessment of environmental surveillance for the detection of poliovirus implementation in the metropolitan districts of South Africa, 2020-2023

**DOI:** 10.11604/pamj.2025.51.58.45463

**Published:** 2025-06-26

**Authors:** Daudi Manyanga, Elizabeth Maseti, Kingsley Mokoena, Thulasizwe Buthelezi, Simangele Mthetwa, Sibongile Mokoena, Ester Khosa-Lesola, Sarah Wanyoike

**Affiliations:** 1 World Health Organization, Inter-Country Support Team Office for East and Southern Africa, Harare, Zimbabwe,; 2 National Department of Health, Pretoria Townlands, Pretoria, South Africa,; 3 World Health Organization, South Africa Office, Tramshed, Pretoria, South Africa

**Keywords:** Polio eradication, wastewater surveillance, poliovirus, environmental surveillance

## Abstract

**Introduction:**

South Africa began using environmental surveillance (ES) in July 2019 to supplement acute flaccid paralysis surveillance. A study is evaluating sites in eight metropolitan health districts for performance improvement and expansion of ES within and across neighbouring countries.

**Methods:**

we conducted a descriptive analysis of secondary ES to detect poliovirus supplemented with qualitative field visits and focused group discussions at 16 ES sites in the eight metropolitan districts of South Africa. The study covered data from January 2020 to December 2023, and tests were conducted to examine the relationship between practices and laboratory results.

**Results:**

in 2021, the National Institute for Communicable Diseases (NICD) Laboratory received 567 ES samples for poliovirus detection, with 97.9% arriving within 72 hours of collection. Monthly sampling increased from 102 (18%) in 2020 to 184 (32.5%) in 2021, showing a 14.5% change with a p-value of 0.0085. There was no statistically significant difference in enterovirus isolation rates between sites trained virtually and in person in the long term, with a 16.5% difference and a p-value of 0.0977. Some ES sites showed high enterovirus isolation rates despite not having specific peak hours, suggesting operational variability in densely populated cities and other areas.

**Conclusion:**

the ES assessment in South Africa has made progress in identifying enteroviruses at all sites. However, irregularities in monthly sampling and peak hours for sample collection need attention. The study suggests using a similar training approach in areas with accessibility challenges and updating guidelines with special consideration for major cities.

## Introduction

In May 1988, during the forty-first World Health Assembly, on the 13^th^ of May, agenda 12, the World Health Organization (WHO) passed resolution WHA41.28, focused on the global eradication of poliomyelitis by 2000 [1]. This resolution followed the achievements made in resolution WHA30.53 of 19^th^ May 1977, which raised the third dose of poliomyelitis vaccine coverage, and the progress made in the Americas, Europe, and the Western Pacific WHO regions towards polio eradication [2]. The initial strategies focused on vaccination and clinical and laboratory surveillance, and they remained the main interventions to reach global polio eradication for more than 36 years. In 2001, after the completion of the primary target for the global polio eradication, the number of polio-endemic countries declined from 125 to 10. These were Nigeria, Niger, Angola and Ethiopia (African Region), Pakistan, Afghanistan, Egypt, Sudan and Somalia (Eastern Mediterranean), and India (Western Pacific) [3]. Interestingly, as of 2024, Afghanistan and Pakistan remained endemic to polio [4,5].

Until 1990, clinical confirmation of poliomyelitis was the primary method of diagnosing the disease when the WHO introduced a global polio laboratory network [6]. In 2003, the concept and procedure of isolating the poliovirus from the environment were documented in the first WHO environmental surveillance guideline and the fourth revision of the laboratory manual [7,8]. In the African Region, environmental surveillance to detect poliovirus began in 2011 in Kano, Nigeria, and three years later in Kenya [9-12]. South Africa initiated environmental surveillance (ES) for poliovirus detection in 2018 in four sites in the Tshwane and Johannesburg cities. Currently, the country has over 26 environmental surveillance sites in all major cities and high-risk areas for the importation of polio outbreaks.

In polio surveillance, acute flaccid paralysis (AFP) surveillance has been the traditional method, and environmental surveillance is used to enhance the sensitivity of the surveillance system [13,14]. Studies conducted in Pakistan and Afghanistan have shown that in areas where the AFP detection sensitivity ranges from 0.5% to 3%, the chances of detecting poliovirus (Sabin) from environmental surveillance range from 34% to 50% [15]. This indicates the potential for environmental surveillance to be 11.3 to 100 times more sensitive than AFP surveillance [15,16]. In this context, implementing ES in South Africa and its neighbouring countries could complement the existing AFP surveillance, which has been shown to have gaps, especially after the COVID-19 pandemic [17,18].

This study evaluates and documents the implementation of Environmental Surveillance (ES) in metropolitan districts of South Africa from January 2020 to December 2023. It focuses on the standard WHO ES performance indicators outlined in the Global Polio Eradication Strategy 2022-26 and the Global Polio Surveillance Action Plan. The study aims to help the country identify and strengthen areas of concern, explore opportunities for integrating the detection of other Vaccine-Preventable Diseases (VPDs) beyond poliomyelitis surveillance, recommend further research, and optimize ES in strategic areas through the deployment of innovations and technologies.

## Methods

**Study design:** we conducted an evaluation of the environmental surveillance sites in the metropolitan districts of South Africa from January 1^st^, 2020, to December 31^st^, 2023. The evaluation involved secondary data analysis collected from the polio laboratory network data on ES and field visits. All 16 ES sites in the cities of Buffalo City (East London), City of Cape Town, Ekurhuleni Metropolitan Municipality (East Rand), City of eThekwini (Durban), City of Johannesburg, Mangaung Municipality (Bloemfontein), Nelson Mandela Bay Metropolitan Municipality (Gqeberha), and City of Tshwane (Pretoria) were included. The field visit teams used a focused discussion guide, which complemented the quantitative data analysis and records reviews.

**Variables and measurements:** the standard global polio surveillance indicators for ES were used in this study and supplemented by qualitative information gathered from various reports [13,19]. Such variables include the proportion of sewage samples that arrive at a WHO-accredited lab ≤3 days of sample collection, enterovirus (EV) detection rate, condition of ES samples receipt at the polio laboratory, and ES samples collected on schedule.

**Inclusion criteria for study cases:** all wastewater samples collected from the validated environmental surveillance sites that met the WHO standards and delivered to the respective polio laboratories from 1^st^ January 2020 to 31^st^ December 2023 in the listed metropolitan districts of South Africa were included in the study. The data source was the field global polio laboratory network ES data shared on a weekly basis and supplemented by field observations. The data may also be retrieved in the global polio eradication initiative database.

**Exclusion criteria for study cases:** any other wastewater samples collection that was not collected for poliovirus analysis purposes or not shared in the Global Polio Laboratory Network database or collected before or after the study period, January 1, 2020, to December 31, 2023, for the metropolitan districts of South Africa. All wastewater samples and those with missing key information, for example, the date of collection, were regarded as “missing” while populating excluded from the respective tables.

## Results

All data for the 16 (100%) environmental surveillance sites for poliovirus detection data were available for the secondary data analysis. However, only 15 (93.7%) of the ES sites were accessed by the evaluation team in the 8 metros. A total of 567 (73.8%) ES samples were collected and delivered to the National Institute for Communicable Diseases (NICD) polio laboratory for analysis during the study period (Table 1). This indicates that many of the ES sites were not following the monthly collection schedule for ES samples as expected. In 2020, only 102 samples (18%) were collected, while most of the samples (32.5%) were collected in 2021. This represents a 14.5 percent change with a p-value of 0.0085 (95% CI ranges from 3.83 to 23.93), showing a statistically significant difference in ES sample collection between the two years. The increase in 2021 was primarily due to the Kwanobuhle, Goudkoppies, Mdantsane, and East Bank wastewater treatment works.

**Table 1:** proportion of environmental surveillance samples collected and the percentage of monthly samples collected by ES sites in metropolitan districts of South Africa from 2020 to 2023

Name of the ES site	Proportion of ES samples collected				Percentage of ES samples collected on schedule monthly				
	2020	2021	2022	2023	2020	2021	2022	2023	Average
Bloemspruit WWTW (n=38)	15.8	28.9	28.9	26.3	50.0	91.7	91.7	83.3	79.2
Borcherds Quarry WWTW (n=34)	17.6	32.4	20.6	29.4	50.0	91.7	58.3	83.3	70.8
Brickfields WWTW (n=32)	12.5	37.5	18.8	31.3	33.3	100.0	50.0	83.3	66.7
Daspoort WWTW (n=51)	33.3	21.6	23.5	21.6	100.0	91.7	100.0	91.7	95.8
East Bank WWTW (n=29)	13.8	41.4	31.0	13.8	33.3	33.3	75.0	33.3	43.8
Erwat Vlakplaats WWTW (n=34)	5.9	32.4	29.4	32.4	16.7	100.0	83.3	91.7	72.9
Gap Northern WWTW (n=42)	38.1	26.2	23.8	11.9	100.0	100.0	83.3	41.7	81.3
Goudkoppies WWTW (n=29)	6.9	37.9	37.9	17.2	16.7	91.7	91.7	41.7	60.4
Hartebeesfontein WWTW (n=48)	29.2	25.0	25.0	20.8	100.0	100.0	100.0	83.3	95.8
Kwanobuhle WWTW (n=29)	6.9	41.4	17.2	34.5	16.7	100.0	41.7	83.3	60.4
KZP Central WWTW (n=35)	14.3	31.4	25.7	28.6	41.7	91.7	75.0	83.3	72.9
KZP Northern WWTW (n=29)	17.2	37.9	13.8	31.0	41.7	91.7	33.3	75.0	60.4
Mdantsane WWTW (n=28)	14.3	42.9	28.6	14.3	33.3	100.0	66.7	33.3	58.3
Rooiwal WWTW (n=33)	9.1	33.3	33.3	24.2	25.0	91.7	91.7	66.7	68.8
Sterkwater WWTW (n=39)	15.4	30.8	25.6	28.2	50.0	100.0	83.3	91.7	81.3
Zandvleit WWTW (n=37)	16.2	35.1	21.6	27.0	50.0	100.0	66.7	83.3	75.0
South Africa overall (n=567)	18.0	32.5	25.2	24.3	47.4	92.2	74.5	71.9	71.5

ES: environmental surveillance; WWTW: wastewater treatment works

In the past four years, the Daspoort wastewater works consistently collected 95.8% of their monthly ES samples, while the East Bank wastewater works only managed to collect 43.8%. This shows a significant 52% difference between the two facilities, with a p-value of less than 0.00001 (95% CI ranges from 31.945 to 68.532). The overall monthly ES sample collection rate for all metropolitan districts' sites was 71.5%.

The time duration between the earliest and latest collection times of ES samples varied across different wastewater treatment plants. Out of the 16 ES sites, the best practices in maintaining consistent collection times were observed at 6 (37.5%) sites: Rooiwal, Sterkwater, Bloemspruit, Hartebeesfontein, KZP Central, and KZP Northern wastewater works (Table 2). Despite the appropriateness of the decided peak hours, all teams maintained a one-hour collection time. The variation between the latest and earliest collections across the ES sites ranged from 0 minutes in Rooiwal wastewater works to 446 minutes (Goudkoppies wastewater works), which is more than 7 hours. About 25% of the ES sites had a time variation of more than 3 hours for the study period, and Gap Northern and Zandvleit wastewater treatment plants did not observe the peak hours consistently. In terms of average time interval, Borcherds Quarry, Gap Northern, Goudkoppies, and Zandvleit wastewater works averaged a variation of more than 3 hours between the latest and earliest collection times.

**Table 2:** distribution of the time intervals between the late and earlier collection times of environmental surveillance samples collected by ES sites in metropolitan districts of South Africa from 2020 to 2023

Name of the ES site	Time interval (late-early time of collection) in minutes					
	2020	2021	2022	2023	Duration range (max -min range)	Average duration (2020-2023)
Bloemspruit WWTW (n=38)	0	30	0	0	30	8
Borcherds Quarry WWTW (n=34)	180	73	179	293	220	181
Brickfields WWTW (n=32)	0	115	70	60	115	61
Daspoort WWTW (n=51)	110	115	60	120	60	101
East Bank WWTW (n=29)	265	55	160	38	227	130
Erwat Vlakplaats WWTW (n=34)	105	60	30	180	150	94
Gap Northern WWTW (n=42)	230	260	231	245	30	242
Goudkoppies WWTW (n=29)	25	471	236	95	446	207
Hartebeesfontein WWTW (n=48)	28	20	23	20	8	23
Kwanobuhle WWTW (n=29)	0	110	45	75	110	58
KZP Central WWTW (n=35)	10	0	67	0	67	19
KZP Northern WWTW (n=29)	48	32	20	46	28	37
Mdantsane WWTW (n=28)	170	156	105	40	130	118
Rooiwal WWTW (n=33)	0	0	0	0	0	0
Sterkwater WWTW (n=39)	0	0	0	30	30	8
Zandvleit WWTW (n=37)	304	185	120	165	184	194
South Africa overall	92	105	84	88	21	92.3

ES: environmental surveillance; WWTW: wastewater treatment works

In the 8 metropolitan districts, 97.9% of ES samples were received within 3 days of collection (Table 3). During the study period in 2023, Mdantsane wastewater works only delivered 75% of its ES samples to the NICD within three days of collection. However, overall, 93.8% of samples collected at the site were delivered within three days and were all in good condition. All ES sites collected over the study period were delivered in good condition at NICD, except for Gap Northern and Sterkwater Gap Northern wastewater works, where 96.2% and 97.9% of their ES samples were received in good condition, respectively. In 2022 and 2023, all samples from the 16 ES sites were delivered in good condition to NICD. In the previous years, 2020 and 2021, 99% of the samples received were in good condition, surpassing the 80% target.

**Table 3:** percentage of environmental surveillance samples received at NICD within 3 days of collection and in good condition by ES sites in metropolitan districts of South Africa from 2020 to 2023

Name of the ES site	Percentage of ES samples reached NICD within 3 days of collection					The percentage of samples arrived in good condition				
	2020	2021	2022	2023	Average	2020	2021	2022	2023	Average
Bloemspruit WWTW (n=38)	100.0	100.0	100.0	100.0	100.0	100.0	100.0	100.0	100.0	100.0
Borcherds Quarry WWTW (n=34)	100.0	100.0	100.0	100.0	100.0	100.0	100.0	100.0	100.0	100.0
Brickfields WWTW (n=32)	100.0	83.3	100.0	80.0	90.8	100.0	100.0	100.0	100.0	100.0
Daspoort WWTW (n=51)	100.0	100.0	100.0	90.9	97.7	100.0	100.0	100.0	100.0	100.0
East Bank WWTW (n=29)	100.0	100.0	88.9	100.0	97.2	100.0	100.0	100.0	100.0	100.0
Erwat Vlakplaats WWTW (n=34)	100.0	100.0	90.0	100.0	97.5	100.0	100.0	100.0	100.0	100.0
Gap Northern WWTW (n=42)	93.8	100.0	100.0	100.0	98.4	93.8	90.9	100.0	100.0	96.2
Goudkoppies WWTW (n=29)	100.0	100.0	100.0	100.0	100.0	100.0	100.0	100.0	100.0	100.0
Hartebeesfontein WWTW (n=48)	100.0	100.0	100.0	100.0	100.0	100.0	100.0	100.0	100.0	100.0
Kwanobuhle WWTW (n=29)	100.0	91.7	100.0	90.0	95.4	100.0	100.0	100.0	100.0	100.0
KZP Central WWTW (n=35)	100.0	100.0	100.0	100.0	100.0	100.0	100.0	100.0	100.0	100.0
KZP Northern WWTW (n=29)	100.0	100.0	100.0	100.0	100.0	100.0	100.0	100.0	100.0	100.0
Mdantsane WWTW (n=28)	100.0	100.0	100.0	75.0	93.8	100.0	100.0	100.0	100.0	100.0
Rooiwal WWTW (n=33)	100.0	100.0	100.0	100.0	100.0	100.0	100.0	100.0	100.0	100.0
Sterkwater WWTW (n=39)	100.0	100.0	100.0	100.0	100.0	100.0	91.7	100.0	100.0	97.9
Zandvleit WWTW (n=37)	100.0	92.3	100.0	90.0	95.6	100.0	100.0	100.0	100.0	100.0
South Africa overall	99.0	97.9	98.6	95.7	97.9	99.0	99.0	100.0	100.0	99.5

ES: environmental surveillance; WWTW: wastewater treatment works; NICD: National Institute for Communicable Diseases

During the evaluation period (2020 to 2023), the overall rate of enterovirus isolation at the 16 ES sites in the metropolitan districts of South Africa ranged from 50% in 2020 to 79.7% in 2022 (Table 4). This represents a 29.7 percentage point change between 2020 and 2022, with a p-value of less than 0.0001 (95% CI ranges from 17.681 to 40.439) and a Chi-square value of 23.778. Variation in enterovirus isolation rates among the ES sites was also observed, with the lowest rate at East Bank wastewater works (39.8%) and the highest rate at Zandvleit wastewater works (90.7%) from 2020 to 2023. This represents a 50.9 percentage point variation between the two sites, with a p-value of less than 0.0001 (95% CI ranges from 28.405 to 67.557) and a Chi-square value of 19.171.

**Table 4:** percentage of enterovirus isolation in environmental surveillance samples collected by ES sites in metropolitan districts of South Africa from 2020 to 2023

Name of the ES site	Percentage of enterovirus isolation							
	2020	2021	2022	2023	Average (2020 - 2023)	Proportion below 50%	Proportion 50% - 80%	Proportion 80% and above
Bloemspruit WWTW (n=38)	0.0	72.7	81.8	50.0	51.5	0.25	0.50	0.25
Borcherds Quarry WWTW (n=34)	83.3	90.9	71.4	80.0	81.9	0.00	0.25	0.75
Brickfields WWTW (n=32)	25.0	66.7	100.0	60.0	63.9	0.25	0.50	0.25
Daspoort WWTW (n=51)	52.9	63.6	83.3	54.6	66.6	0.00	0.75	0.25
East Bank WWTW (n=29)	0.0	41.7	77.8	75.0	39.8	0.50	0.50	0.00
Erwat Vlakplaats WWTW (n=34)	50.0	63.6	70.0	81.8	61.2	0.00	0.75	0.25
Gap Northern WWTW (n=42)	62.5	45.5	100.0	60.0	69.3	0.25	0.50	0.25
Goudkoppies WWTW (n=29)	50.0	81.8	72.7	100.0	68.2	0.00	0.50	0.50
Hartebeesfontein WWTW (n=48)	57.1	91.7	83.3	70.0	77.4	0.00	0.50	0.50
Kwanobuhle WWTW (n=29)	50.0	66.7	100.0	70.0	72.2	0.00	0.75	0.25
KZP Central WWTW (n=35)	40.0	72.7	66.7	60.0	59.8	0.25	0.75	0.00
KZP Northern WWTW (n=29)	60.0	81.8	100.0	66.7	80.6	0.00	0.50	0.50
Mdantsane WWTW (n=28)	25.0	75.0	75.0	50.0	58.3	0.25	0.75	0.00
Rooiwal WWTW (n=33)	66.7	45.5	45.5	50.0	52.5	0.50	0.50	0.00
Sterkwater WWTW (n=39)	16.7	83.3	90.0	63.6	63.3	0.25	0.25	0.50
Zandvleit WWTW (n=37)	100.0	84.6	87.5	90.0	90.7	0.00	0.00	1.00
South Africa overall	50.0	71.1	79.7	67.4	66.9	0.16	0.52	0.33

ES: environmental surveillance; WWTW: wastewater treatment works

We observed that three (18.75%) of the ES sites achieved an average EV isolation rate of 80% or more during the evaluation period. Only 1 site (East Bank wastewater works) had an EV isolation rate below 50%, while the majority had an average EV isolation rate between 50% and 80%. Interestingly, Zandvleit wastewater works achieved over 80% EV isolation each year during the evaluation period. Overall, 50% of the ES sites in the metropolitan district of South Africa achieved an EV isolation rate of 50% or more for the study period. Evaluating the 6 ES sites that were facilitated by physical visitation, Gap-Northern, Daspoort, Erwat Vlakplaats, Goudkoppies, Hartebeesfontein, and Rooiwal wastewater treatment works, and the remaining 10 (Bloemspruit, Borcherds Quarry, Brickfields, East Bank, Kwanobuhle, KZP-Central, KZP Northern, Mdantsane, Sterkwater, and Zandvleit wastewater works) that were facilitated remotely (Table 5). In terms of the 2020 performance, the ES sites with physical visitation and training had a high EV isolation rate averaging 56.5% and no sites had below 50% isolation margin level while the ES sites supported virtually with no initial physical visitation had EV isolation rate averaged at 40% and 60% of the sites had EV isolation rate below the 50% margin. This indicated a 16.5 percentage difference, p-value of 0.0977 (95% CI ranges from -2.822 to 34.155), and Chi-square value of 2.74, which all suggest no statistical significance.

**Table 5:** comparison of EV isolation rate in environmental surveillance samples collected by ES sites that received physical and virtual training in metropolitan districts of South Africa from 2020 to 2023

Name of the ES site	Percentage of enterovirus isolation					Percentage of ES samples collected on schedule, monthly				
	2020	2021	2022	2023	Average	2020	2021	2022	2023	Average
Face-to-face trained ES sites Daspoort WWTW (n=51)	52.9	63.6	83.3	54.6	63.6	100.0	91.7	100.0	91.7	95.8
Erwat Vlakplaats WWTW (n=34)	50.0	63.6	70.0	81.8	66.4	16.7	100.0	83.3	91.7	72.9
Gap Northern WWTW (n=42)	62.5	45.5	100.0	60.0	67.0	100.0	100.0	83.3	41.7	81.3
Goudkoppies WWTW (n=29)	50.0	81.8	72.7	100.0	76.1	16.7	91.7	91.7	41.7	60.4
Hartebeesfontein WWTW (n=48)	57.1	91.7	83.3	70.0	75.5	100.0	100.0	100.0	83.3	95.8
Rooiwal WWTW (n=33)	66.7	45.5	45.5	50.0	51.9	25.0	91.7	91.7	66.7	68.8
Average	56.5	65.3	75.8	69.4	66.8	59.7	95.8	91.7	69.4	79.2
Virtually trained ES sites Bloemspruit WWTW (n=38)	0.0	72.7	81.8	50.0	51.1	50.0	91.7	91.7	83.3	79.2
Borcherds Quarry WWTW (n=34)	83.3	90.9	71.4	80.0	81.4	50.0	91.7	58.3	83.3	70.8
Brickfields WWTW (n=32)	25.0	66.7	100.0	60.0	62.9	33.3	100.0	50.0	83.3	66.7
East Bank WWTW (n=29)	0.0	41.7	77.8	75.0	48.6	33.3	33.3	75.0	33.3	43.8
Kwanobuhle WWTW (n=29)	50.0	66.7	100.0	70.0	71.7	16.7	100.0	41.7	83.3	60.4
KZP Central WWTW (n=35)	40.0	72.7	66.7	60.0	59.9	41.7	91.7	75.0	83.3	72.9
KZP Northern WWTW (n=29)	60.0	81.8	100.0	66.7	77.1	41.7	91.7	33.3	75.0	60.4
Mdantsane WWTW (n=28)	25.0	75.0	75.0	50.0	56.3	33.3	100.0	66.7	33.3	58.3
Sterkwater WWTW (n=39)	16.7	83.3	90.0	63.6	63.4	50.0	100.0	83.3	91.7	81.3
Zandvleit WWTW (n=37)	100.0	84.6	87.5	90.0	90.5	50.0	100.0	66.7	83.3	75.0
Average	40.0	73.6	85.0	66.5	66.3	40.0	90.0	64.2	73.3	66.9

ES: environmental surveillance; WWTW: wastewater treatment works; EV: enterovirus

In 2021, after one year of practical experience, the EV isolation rate improved in ES sites that were capacitated virtually from 40% in 2020 to 73.6%, indicating a 33.6-point percentage change, a p-value of less than 0.0003 (95% CI ranges from 17.063 to 48.098) and Chi-square value of 16.542, which is statistically significant. In the evaluation period, the year with a maximum of 2022 for ES sites that were capacitated virtually and had an EV isolation rate of 85% while the physically capacitated ES sites had a 75.8% EV isolation rate. Again, though the point percentage change was 9.2 for the two groups of ES sites, the p-value was 0.165 (95% CI ranges from -3.789 to 22.366) and the Chi-square value was 1.922, which all indicated no significant variation.

## Discussion

The evaluation revealed weaknesses in the monthly collection of ES samples in the metropolitan districts' ES sites. During field visits, discussions highlighted a shortage of human resources contributing to the variations in practices, such as the involvement of different personnel in the sampling process, ranging from wastewater treatment works staff and field researchers to health district staff, including environmental health practitioners. While regularity in sample collection was observed in areas where the collectors were from the ES sites, challenges were reported in transporting the samples from the site to the NICD. It was evident that the decline in collection in 2020 was primarily related to the COVID-19 pandemic, but the further decline in 2023 was not well explained or explored. Additionally, it was observed that private facilities had more regularity in sample collection compared to public or parastatal facilities. This could be attributed to the initial difficulties in engaging with wastewater works in private facilities that led to high management decision-making, which was sustained in terms of performance compared to others. It is evident that due to infrequent samplings, although the ES system was sensitive, there is a possibility of missing transmission that could have occurred during the uncollected period. Among other measures, the country should focus on improving and adhering to monthly collection practices.

We recognize that our environmental sampling guideline requires strict adherence to peak hours, which is crucial for isolating viruses. Upon evaluation, we observed consistent variations in sampling times at some sites, with more than a 3-hour difference. Out of the 4 sites with more than 3 hours difference, 2 (50%) were consistently top performers in isolating enteric viruses. Interestingly, one site that was consistently sampled on time had a poor EV isolation rate over 2 years within the 4-year evaluation. This suggests the need for careful observation of wastewater treatment plants to determine the appropriate peak hours. It's also possible that a site may have multiple peak hours, allowing for increased detection of various polioviruses based on different activities throughout the day in urban areas. For example, daytime or transit populations may contribute to EV detections in delayed sample collection, rather than just a few individuals using the sewage system in the morning.

Most ES samples were delivered to NICD within 3 days. However, surprisingly, 75% of the samples from the Mdantsane wastewater works in 2023 were not delivered within 3 days. On the other hand, in East Bank wastewater works, 88.9% of ES samples in 2022 and in Brickfield, 83.3% of ES samples were delivered within 3 days. This indicates operational issues in the preparation and collection of samples by the couriers in East London and Nelson Mandela Bay, which may lead to some ES samples not being collected in the future. Nonetheless, all ES samples from the mentioned sites were received in good condition. The EV isolation rate from the samples was low in East Bank wastewater works and relatively low in Mdantsane wastewater works. Regarding the sample condition, Gap Northern wastewater works scored low for 2020 and 2021, even though the site is near the NICD within the city of Johannesburg. This indicates possible sample transportation challenges that were not discussed in detail.

We also noted that, in a situation where physical accessibility may not be possible, virtual capacity building is advised to initiate the ES site may be possible with close remote follow-up. We noted that of the ten sites initiated with remote support in the first year, 60% had low EV isolation rates ranging from 0% to 40%, and 4 ES sites achieved between 50% and 100%. This indicates a wide variation among virtually capacitated ES sites, while the physical capacitated EV isolation was more than 50% in all sites and ranged between 50% and 66%. In 2023, all sites had achieved an EV isolation rate of more than 50% 2 (33.3%) of the physical visits had achieved more than 80% and above the EV isolation rate. Furthermore, 2 (20%) of the virtual capacitation ES sites achieved 80% and above EV isolation rates. These are impressive results, but still indicate the importance of physical capacity in comparison with virtual capacity building regarding ES implementation for high-quality results.

**Study limitations:** the study did not assess environmental surveillance in 2024 because the year had not concluded. Therefore, it was not possible to evaluate any samples that were collected but not delivered to the NICD. Furthermore, we only assessed environmental surveillance for poliovirus detection and not for other pathogens.

## Conclusion

The evaluation of environmental surveillance (ES) in metropolitan districts of South Africa has shown significant progress in terms of isolating enteroviruses (EV) from all sites, both before and after the COVID-19 pandemic lockdown restrictions. However, there are gaps in adhering to the monthly collection schedule and addressing peak hours for sample collection. It is important to correct these issues at current sites and optimize other strategic ES sites. The evaluation suggests that similar approaches for initiating ES sites remotely can be used in countries where accessibility challenges exist in order to improve the sensitivity of the surveillance system for detecting polioviruses. The experience of South Africa also highlights the need to consider different peak hours for ES sites in cities, as they may have multiple peak hours.

### What is known about this topic

- Environmental surveillance is an essential tool for poliovirus monitoring in the WHO African region as well as globally for polio eradication;
- Environmental surveillance helps to track the spread of enteroviruses and assess the extent and duration of poliovirus outbreaks in specific populations;
- In certain countries, wild polioviruses or circulating vaccine-derived polioviruses (e.g. Botswana, Uganda, etc.) have been detected in the environment even when no cases of acute flaccid paralysis (AFP) have been reported; environmental surveillance is more sensitive than acute flaccid paralysis.

### What this study adds

- The study emphasizes the need to optimize and scale up ES in South Africa and other countries to enhance the sensitivity in detecting and localizing transmissions;
- The study also suggests the potential for multiple peak hours for ES sample collection, which could lead to higher detection of polioviruses;
- The study highlights the need to use the South African experience to initiate environmental surveillance for poliovirus remotely in accessibility-challenged areas, with possible integration with other vaccine-preventable disease surveillance efforts.

## References

[1] World Health Organization (1988). Global eradication of poliomyelitis by the year 2000.

[2] World Health Organization (1976). WHA29.63: Expanded programme on immunization.

[3] World Health Organization (2002). Progress towards the global eradication of poliomyelitis, 2001. Wkly Epidemiol Rec.

[4] Mbaeyi C, Ul Haq A, Safdar RM, Khan Z, Corkum M, Henderson E (2024). Progress Toward Poliomyelitis Eradication-Pakistan, January 2023-June 2024. MMWR Morb Mortal Wkly Rep.

[5] Africa Regional Commission for the Certification of Poliomyelitis Eradication (2020). Certifying the interruption of wild poliovirus transmission in the WHO African region on the turbulent journey to a polio-free world. Lancet Glob Health.

[6] World Health Organization (1990). Manual for the virological investigation of poliomyelitis.

[7] World Health Organization (2003). Guidelines for environmental surveillance of poliovirus circulation.

[8] World Health Organization (2004). Polio laboratory manual.

[9] Gumede N, Okeibunor J, Diop O, Baba M, Barnor J, Mbaye S (2018). Progress on the Implementation of Environmental Surveillance in the African Region, 2011-2016. J Immunol Sci.

[10] Hamisu AW, Johnson TM, Craig K, Mkanda P, Banda R, Tegegne SG (2016). Strategies for Improving Polio Surveillance Performance in the Security-Challenged Nigerian States of Adamawa, Borno, and Yobe During 2009-2014. J Infect Dis.

[11] Tushabe P, Bwogi J, Eliku JP, Aine F, Birungi M, Gaizi J (2023). Environmental surveillance detects circulating vaccine-derived poliovirus type 2 that was undetected by acute flaccid paralysis surveillance in 2021 in Uganda. Arch Virol.

[12] Eboh VA, Makam JK, Chitale RA, Mbaeyi C, Jorba J, Ehrhardt D (2018). Notes from the Field: Widespread Transmission of Circulating Vaccine-Derived Poliovirus Identified by Environmental Surveillance and Immunization Response-Horn of Africa, 2017-2018. MMWR Morb Mortal Wkly Rep.

[13] World Health Organization (2022). Global Polio Surveillance Action Plan 2022-2024.

[14] Centers for Disease Control and Prevention (2021). Epidemiology and Prevention of Vaccine-Preventable Diseases: Chapter 18: Poliomyelitis.

[15] Kroiss SJ, Ahmadzai M, Ahmed J, Alam MM, Chabot-Couture G, Famulare M (2018). Assessing the sensitivity of the polio environmental surveillance system. PLoS One.

[16] Lodder WJ, Buisman AM, Rutjes SA, Heijne JC, Teunis PF, de Roda Husman AM (2012). Feasibility of quantitative environmental surveillance in poliovirus eradication strategies. Appl Environ Microbiol.

[17] Manyanga D, Masvikeni B, Kuloba M, Byabamazima C, Daniel F (2021). Early effects of the COVID-19 pandemic on the acute flaccid paralysis surveillance in East and Southern African countries. Pan African Medical Journal.

[18] Manyanga D, Byabamazima C, Masvikeni B, Daniel F (2020). Assessment of acute flaccid paralysis surveillance performance in East and Southern African countries 2012 - 2019. Pan African Medical Journal.

[19] World Health Organization, Global Polio Eradication Initiative (2023). Field guidance for the implementation of environmental surveillance for poliovirus.

